# Residual β-cell function is associated with continuous glucose monitoring metrics in patients with type 1 diabetes mellitus

**DOI:** 10.3389/fendo.2026.1891725

**Published:** 2026-08-06

**Authors:** Jing Kang, Qinyan Huang, Yuanshuang Jiang, Yan Chen

**Affiliations:** Department of Endocrinology, The Second Hospital of Jilin University, Changchun, China

**Keywords:** continuous glucose monitoring, fasting C-peptide, residual β-cell function, time in range, type 1 diabetes mellitus

## Abstract

**Background:**

To evaluate the relationship between residual islet β-cell function assessed by fasting C-peptide (FCP) and continuous glucose monitoring (CGM) metrics in patients with type 1 diabetes mellitus (T1DM), and to clarify the impact of residual islet β-cell function on glycemic control in T1DM.

**Methods:**

A retrospective study was conducted including 112 patients with T1DM [68 classic T1DM, 44 latent autoimmune diabetes in adults (LADA)] hospitalized from January 2023 to December 2025. All patients wore CGM devices for ≥7 days. Participants were stratified into four groups by FCP quartiles. Spearman correlation, restricted cubic spline (RCS) regression, and multivariable linear regression with progressive adjustment for confounders were used to evaluate associations between FCP and CGM-derived metrics, including time in range (TIR), time above range (TAR), and mean glucose (MG). Subgroup and interaction analyses were performed by diabetes subtype. Sensitivity analyses included quartile-based categorization, exclusion of LADA patients, and exclusion of those with diabetes duration ≥10 years.

**Results:**

In this retrospective study, higher FCP levels were significantly associated with favorable CGM-derived metrics, including increased TIR, decreased TAR, and lower MG (all *P* < 0.001). These associations persisted after multivariable adjustment for sex, age, disease duration, BMI, insulin dosage, and glucose coefficient of variation (CV). RCS analysis revealed significant nonlinear dose-response relationships, with pronounced glycemic improvements at lower FCP concentrations and plateau effects at higher levels. LADA patients exhibited higher FCP levels, longer TIR, and lower TAR compared with classic T1DM (all *P* < 0.05). Subgroup analyses demonstrated consistent FCP-CGM associations in both subtypes without significant interaction (all *P* for interaction > 0.05). Sensitivity analyses confirmed robust associations after excluding LADA patients or those with disease duration ≥10 years. Shorter disease duration and lower daily insulin requirements correlated with higher FCP levels.

**Conclusion:**

Residual β-cell function is independently associated with improved CGM-derived metrics (increased TIR, decreased TAR, and lower MG) in both classic T1DM and LADA. Preserved C-peptide secretion correlates with shorter disease duration and lower daily insulin requirements. These findings highlight the importance of protecting residual β-cell function to achieve glycemic stability and reduce exogenous insulin dependence in T1DM.

## 1 Introduction

Type 1 diabetes mellitus (T1DM) is characterized by autoimmune-mediated rapid destruction of β-cell mass (1), resulting in absolute insulin deficiency and necessitating lifelong insulin replacement therapy. According to the 11th edition of the International Diabetes Federation (IDF) Diabetes Atlas, an estimated 589 million adults aged 20–79 years were living with diabetes worldwide in 2024, among whom approximately 9.15 million had been diagnosed with clinical T1DM (2). Notably, in 2024, roughly 30,000 T1DM-related deaths occurred among undiagnosed individuals under 25 years of age who succumbed within 12 months of symptom onset. The substantial mortality attributable to T1DM complications underscores the critical urgency of developing effective therapeutic strategies to delay disease onset.

In most patients with T1DM, insulin secretion declines significantly with prolonged disease duration; however, residual insulin secretion persists. Preservation of long-term residual islet β-cell function in T1DM patients can significantly reduce the risk of severe hypoglycemia (3) and lower the incidence of complications (4, 5). Therefore, residual islet β-cell function is closely associated with prognosis in T1DM, and interventions aimed at protecting limited β-cell function during the early stage of the disease may confer meaningful and durable health benefits. C-peptide and insulin are both cleavage products of proinsulin. Compared with insulin, C-peptide exhibits a slower metabolic clearance rate and is considered a marker of β-cell reserve function and endogenous insulin secretion (6, 7), particularly in T1DM, where C-peptide serves as an excellent marker for assessing residual β-cell function. In the Diabetes Control and Complications Trial (DCCT), patients with T1DM receiving intensive therapy who maintained sustained C-peptide secretion demonstrated better glycemic control, reduced glycated hemoglobin (HbA1c), and decreased insulin requirements (8). C-peptide detection methods include serum random C-peptide, fasting C-peptide (FCP), and stimulated C-peptide (9). Stimulated serum C-peptide testing following provocative challenge is superior to random C-peptide, fasting C-peptide, and urinary C-peptide assessment. However, owing to the stringent requirements and practical inconvenience of provocative testing, even random or FCP levels retain clinical significance and can be utilized to evaluate residual islet β-cell function (10).

Due to pronounced glycemic variability in patients with T1DM, HbA1c alone does not accurately reflect true glycemic conditions. The advent of continuous glucose monitoring (CGM) technology enables data acquisition at 1- to 5-minute intervals, facilitating comprehensive capture of glycemic fluctuations, asymptomatic hypoglycemic events, and hyperglycemic excursions (11). CGM-derived metrics, notably time in range (TIR), address the inherent limitations of HbA1c while demonstrating robust correlation with this established parameter and predictive value for diabetes-related complications, thereby substantially overcoming the shortcomings of HbA1c. The 2020 Standards of Medical Care in Diabetes, published by the American Diabetes Association (ADA), formally established the pivotal role of CGM-derived metrics in comprehensive glycemic assessment. A real-world retrospective cross-sectional study evaluating the association between chronic complications and TIR in T1DM demonstrated that the incidence of any microvascular complication, diabetic retinopathy, diabetic nephropathy, and cerebrovascular accident decreased progressively across ascending TIR quartiles (12). Studies have also shown that persistent C-peptide secretion is associated with favorable CGM metrics in adults with T1DM (13).

Given the established importance of residual islet β-cell function and TIR in maintaining glycemic homeostasis and mitigating complications in patients with T1DM, this study aims to evaluate residual β-cell function through FCP measurement and to investigate its correlations with CGM-derived metrics, including TIR, time below range (TBR), time above range (TAR), and glucose coefficient of variation (CV), thereby further elucidating the clinical significance of preserving islet β-cell function in the management of T1DM.

## 2 Methods

### 2.1 Study design and participants

**Figure 1:**
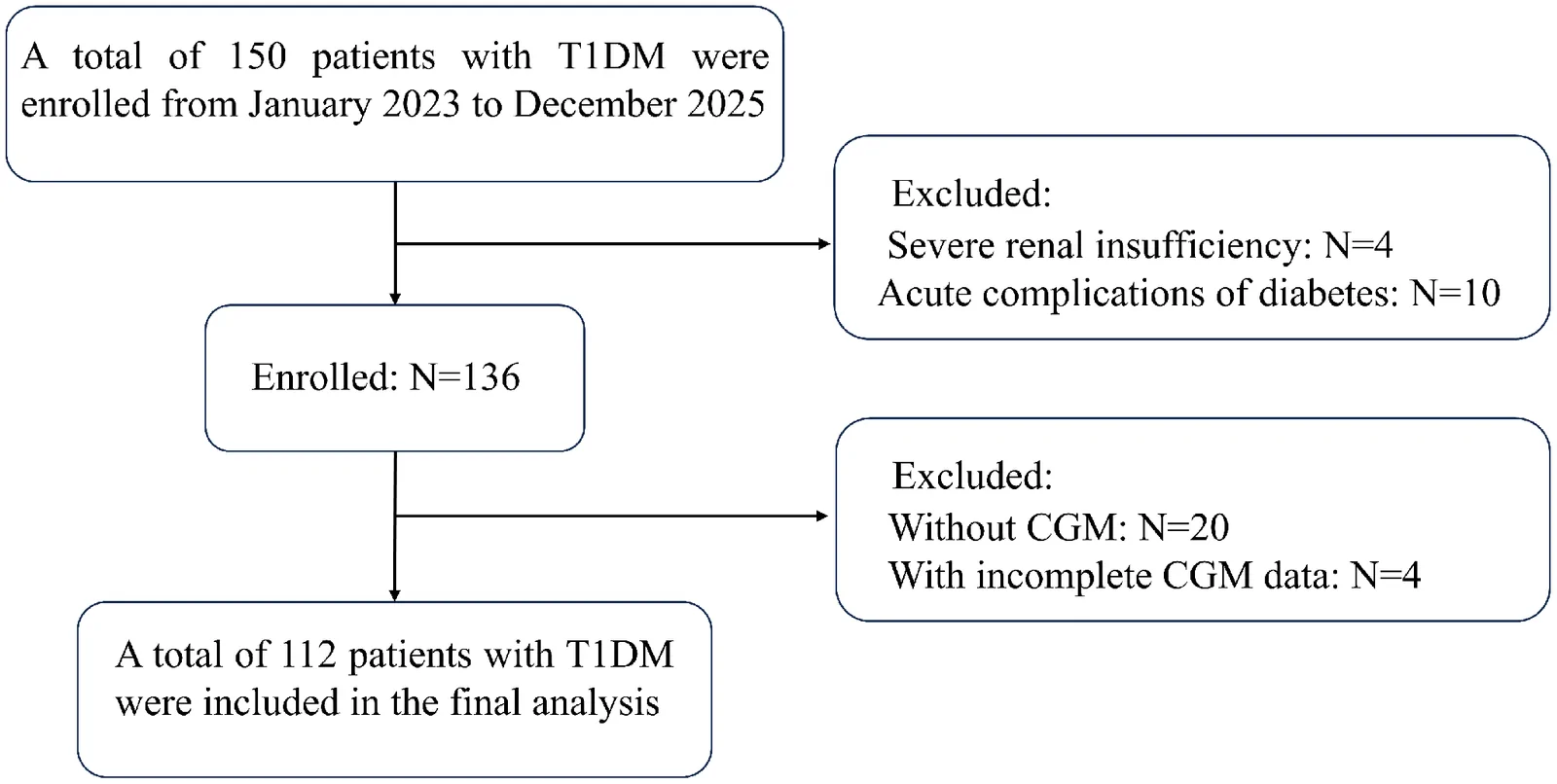
Flow chart of this study.

This retrospective study enrolled patients with T1DM hospitalized in the Department of Endocrinology at the Second Hospital of Jilin University between January 2023 and December 2025. Inclusion criteria were as follows (1): diagnosis of T1DM according to the 2025 ADA Standards of Care, encompassing classic T1DM and latent autoimmune diabetes in adults (LADA) (14). T1DM was diagnosed by the presence of islet autoantibodies (glutamic acid decarboxylase autoantibody [GADA], islet antigen 2 autoantibody [IA-2A], zinc transporter 8 [ZnT8], and/or insulin autoantibodies [IAA]) plus meeting diabetes criteria. LADA was defined as onset at ≥18 years, positive islet autoantibodies, and insulin independence for ≥6 months. Classic T1DM was defined as absolute insulin deficiency (low/undetectable C-peptide) requiring immediate insulin therapy; (2) CGM wear for ≥7 days with complete data (valid recordings >70%); and (3) treatment with continuous subcutaneous insulin infusion via insulin pump alone, using rapid-acting insulin analogs. Exclusion criteria comprised: (1) uncertain diabetes classification or negative pancreatic autoantibodies; (2) severe hepatic or renal insufficiency (estimated glomerular filtration rate <30 mL/min/1.73 m² or alanine aminotransferase >3 times the upper limit of normal); (3) recent acute infection or surgery; (4) pregnancy or lactation; (5) incomplete CGM data or wear duration <7 days; and (6) presence of acute diabetes complications, including diabetic ketoacidosis or hyperosmolar hyperglycemic state. A total of 150 patients with T1DM were screened. Of these, 4 were excluded for severe renal insufficiency, 10 for acute diabetes complications, and 24 for absence of CGM wear or incomplete CGM data. Ultimately, 112 patients were included in the final analysis (**Figure 1**). This study adhered to the principles of the Declaration of Helsinki and was approved by the Medical Ethics Committee of the Second Hospital of Jilin University (approval number: [2026] Research Review No. 107). Informed consent was waived due to the retrospective nature of the study.

### 2.2 Data collection

Demographic and clinical data, including sex, age, body mass index (BMI), disease duration, and daily insulin dosage, were extracted from the electronic medical record system. Relevant laboratory findings were also retrieved. Patients fasted for at least 8 hours following admission, and venous blood samples were collected in the early morning. The following parameters were measured: fasting blood glucose (FBG) HbA1c, FCP, 2-hour postprandial C-peptide (2hCP), islet autoantibodies (IA-2A, IAA, GADA), thyroid peroxidase antibody (TPOAb), triglycerides (TG), total cholesterol (TC), high-density lipoprotein cholesterol (HDL-C), low-density lipoprotein cholesterol (LDL-C), alanine aminotransferase (ALT), aspartate aminotransferase (AST), blood urea nitrogen (BUN), serum creatinine (Scr), uric acid (UA), and estimated glomerular filtration rate (eGFR). FCP was measured using Beckman Coulter reagents on a Beckman Coulter DXI800 fully automated chemiluminescence immunoassay analyzer. The assay principle was based on chemiluminescence immunoassay with a two-site sandwich immunoassay format. Briefly, fasting venous blood samples were collected, and serum was separated and quantitatively analyzed according to the manufacturer’s standard operating procedures. The analytical sensitivity was 0.01 ng/mL. As not all patients underwent systematic screening for diabetic microvascular and macrovascular complications, complications were not incorporated into this study.

### 2.3 CGM-derived metrics

All patients received continuous subcutaneous insulin infusion via insulin pump and wore the iPro2 professional retrospective CGM system (Medtronic, USA). The sensor was inserted into the subcutaneous tissue of the anterior abdominal wall, with glucose data recorded at 5-minute intervals over 7 consecutive days. A minimum of four daily fingerstick blood glucose calibrations were performed. Data were subsequently exported, and the first 7 days of data after admission were selected for analysis using CareLink iPro software. Core CGM metrics were defined in accordance with the 2019 Advanced Technologies & Treatments for Diabetes (ATTD) International Consensus: TIR, defined as the percentage of time with sensor glucose levels between 3.9 and 10.0 mmol/L (target >70%); TAR, defined as the percentage of time with sensor glucose levels >10.0 mmol/L; TBR, defined as the percentage of time with sensor glucose levels <3.9 mmol/L; CV, calculated as (standard deviation of glucose/mean glucose) × 100%; and mean glucose (MG), calculated as the arithmetic mean of all sensor readings.

### 2.4 Statistical analysis

Continuous variables with a normal distribution were expressed as mean ± SD, non-normally distributed variables as median (interquartile range) [M (IQR)], and categorical variables as frequency (percentage) [n (%)]. Normality was assessed using the Shapiro-Wilk test. FCP was divided into four groups based on quartile values: Q1 (<0.01 ng/mL), Q2 (0.01–0.09 ng/mL), Q3 (0.09–0.37 ng/mL), and Q4 (>0.37 ng/mL). For patient characteristics across the FCP quartiles, intergroup comparisons were performed using one-way analysis of variance (ANOVA) or the Kruskal-Wallis H test, with *post-hoc* pairwise comparisons by Tukey’s honestly significant difference method for continuous variables. For clinical characteristics comparisons between the LADA and classic T1DM subgroups, the independent-samples t-test or Mann-Whitney U test was applied for continuous variables. Categorical variables were compared using the χ² test or Fisher’s exact test as appropriate. Spearman correlation analysis was used to assess the correlations between FCP and HbA1c, TIR, TAR, MG, CV, daily insulin dose, diabetes duration, and age. To further explore the potential non-linear dose-response relationships between FCP and CGM metrics (TIR, TAR, and MG), restricted cubic spline (RCS) regression models were fitted with three knots placed at default percentiles according to Harrell’s recommendations. Wald χ² tests were utilized to evaluate the significance of overall and non-linear associations. To evaluate the independent effect of FCP on CGM-derived metrics (TIR, TAR, and MG) and quantify the average associations, multivariable linear regression models were constructed: Model 1, crude analysis; Model 2, adjusted for sex, age, diabetes duration, and BMI; Model 3, additionally adjusted for daily insulin dose; and Model 4, further adjusted for glucose CV. The RCS models were adjusted for the same covariates as in Model 4. To address potential clinical heterogeneity, subgroup analyses and restricted cubic spline comparisons were performed according to diabetes subtype (LADA vs. classic T1DM). Testing for interaction was conducted by including a multiplicative interaction term (FCP * diabetes subtype) in the multivariable models. Finally, to verify the robustness of our primary findings, three multi-faceted sensitivity analyses were performed using the fully adjusted model: (1) re-analyzing FCP as a quartile-based categorical variable, where the linear trend across FCP categories (P for trend) was evaluated by assigning the median value of each FCP quartile as a continuous variable in the regression models; (2) entirely excluding LADA patients to restrict the analysis to the classic T1DM sub-cohort; and (3) entirely excluding patients with a diabetes duration ≥ 10 years to eliminate potential survivor bias.

All statistical analyses were performed using SPSS 24.0 (IBM, USA), Origin 2021 (OriginLab, USA), and R software version 4.3.0 (R Foundation for Statistical Computing, Vienna, Austria). Two-sided tests were used, and *P* < 0.05 was considered statistically significant.

## 3 Results

### 3.1 Patient characteristics

A total of 112 patients with T1DM (classic T1DM, n=68; LADA, n=44) were included in the final analysis, with a mean age of 41 ± 18 years and 49.1% were male. According to FCP quartiles, patients were divided into four groups: Q1 (<0.01 ng/mL, n = 27), Q2 (0.01–0.09 ng/mL, n = 28), Q3 (0.09–0.37 ng/mL, n = 28), and Q4 (>0.37 ng/mL, n = 29). Baseline characteristics across the four groups are presented in **Table 1**. No significant differences were observed in sex distribution, age, or BMI (all *P* > 0.05). Disease duration differed significantly among the four groups, with shorter duration observed in patients with higher FCP levels (*P* < 0.001). Regarding glycemic parameters, although no significant differences were found in FBG (*P* = 0.421) or HbA1c (*P* = 0.329), CGM-derived metrics including TIR, TAR, and MG showed significant differences. Specifically, higher FCP levels were associated with increased TIR, decreased TAR, lower MG, and reduced daily insulin requirements (all *P* < 0.05). Additionally, LDL-C and UA levels were higher, and more patients were co-positive for IA-2A and GADA in Q4 compared with the other three groups (*P* < 0.05), (*P* < 0.05), while no significant intergroup differences were observed for the remaining biochemical parameters.

**Table 1:** Patient characteristics.

Characteristic	Q1	Q2	Q3	Q4	*P*
Participants (n)	27	28	28	29	–
LADA (%)	18.5%	35.7%	35.7%	65.5%	0.004
FCP (ng/mL)	0.00 (0.00, 0.00)	0.01(0.01, 0.04)	0.23 (0.15, 0.30)	0.55 (0.46, 0.71)	<0.001
Male sex (%)	44.4	50	50	51.7	0.954
Age (years)	43.3 ± 18.28	44.11 ± 20.85	37.07 ± 19.84	41.28 ± 16.05	0.509
BMI (kg/m^2^)	22.09 ± 2.73	21.81 ± 3.48	20.80 ± 4.09	21.92 ± 2.89	0.472
Duration of T1DM (years)	11.26 ± 5.87	8.31 ± 7.14	4.93 ± 5.85	2.72 ± 5.03	<0.001
TIR (%)	30.74 ± 16.59	35.25 ± 19.10	50.75 ± 23.72	64.72 ± 17.92	<0.001
TAR (%)	68.44 ± 17.44	63.64 ± 19.84	48.61 ± 24.05	34.76 ± 18.19	<0.001
TBR (%)	0 (0, 1)	0 (0, 2)	0 (0, 1)	0 (0, 1)	0.337
Glucose CV (%)	30.2 ± 6.16	28.57 ± 7.87	27.92 ± 6.89	27.25 ± 6.39	0.424
MG (mmol/L)	12.33 ± 1.98	11.73 ± 2.06	10.36 ± 2.04	9.26 ± 1.41	<0.001
HbA1c (%)	8.82 ± 1.91	9.26 ± 1.80	9.72 ± 2.94	9.92 ± 2.66	0.329
FBG (mmol/L)	13.80 ± 5.28	12.19 ± 4.51	13.10 ± 4.33	11.82 ± 5.21	0.421
Daily insulin dose (units/day)	37.96 ± 9.12	37.39 ± 8.63	29.21 ± 12.07	24.79 ± 7.60	<0.001
IA-2A (U/mL)	5.39 (5.12, 5.87)	5.15 (4.75, 7.62)	5.47 (4.92, 46.95)	8.0 (4.83, 78.0)	0.493
IAA (U/mL)	6.34 (5.38, 7.26)	16.15 (6.03, 121)	7.16 (5.45, 10.02)	8.96 (6.3, 19.4)	0.296
GADA (U/mL)	16.27 (8.25, 80.63)	59.7 (10.2, 280)	261.5 (52.3, 280)	55 (35.3, 207)	0.008
IA-2A+IAA (n)	0	1	1	2	0.714
IA-2A+GADA (n)	1	2	5	10	0.003
IAA+GADA (n)	2	4	3	4	0.836
IA-2A+IAA+GADA (n)	0	1	1	2	0.714
TPOAb (U/mL)	20.95 (12.7, 108.13)	19.8 (11.83, 110.23)	22.15 (12.4, 231.75)	15.1 (11.7, 63.5)	0.097
TG (mmol/L)	1.32 (1.09, 1.36)	1.28 (0.96, 1.53)	1.16 (0.73, 1.57)	1.12 (0.81, 1.6)	0.932
TC (mmol/L)	5.18 ± 0.94	4.63 ± 1.0	5.43 ± 1.56	5.48 ± 2.02	0.154
LDL-C (mmol/L)	2.98 ± 0.63	2.55 ± 0.74	2.91 ± 1.10	3.30 ± 1.08	0.034
HDL-C (mmol/L)	1.52 ± 0.30	1.42 ± 0.38	1.47 ± 0.48	1.36 ± 0.52	0.582
ALT (U/L)	13 (9.75, 20.25)	15 (11, 20.75)	15 (11, 24)	15.5 (13, 26.75)	0.444
AST (U/L)	16 (13.75, 21)	18 (14.25, 20.75)	19 (13, 27)	17 (15.75, 27)	0.800
BUN (mmol/L)	5.36 ± 1.53	6.51 ± 3.31	5.47 ± 2.11	6.51 ± 3.90	0.278
Scr (umol/L)	68.74 ± 44.22	64.69 ± 24.39	52.24 ± 13.69	64.81 ± 20.37	0.163
UA (umol/L)	225 (196.7, 298.7)	273 (208.5, 346.7)	270 (239, 333)	312.5 (264.2, 398.5)	0.032
eGFR (mL/min)	110.6 (96.3, 131.5)	111.1 (94.9, 128.3)	120 (100.9, 137.5)	115.9 (103.1, 124.9)	0.317

Data are presented as mean ± SD, median (IQR), or n (%). Values of fasting C-peptide are defined as Q1 (<0.01 ng/mL), Q2 (0.01–0.09 ng/mL), Q3 (0.09–0.37 ng/mL), and Q4 (>0.37 ng/mL). ALT, alanine aminotransferase; AST, aspartate aminotransferase; BUN, blood urea nitrogen; CV, coefficient of variation; eGFR, estimated glomerular filtration rate; FBG, fasting blood glucose; FCP, fasting C-peptide, GADA, glutamic acid decarboxylase antibody; HbA1c, glycated hemoglobin; HDL-C, high-density lipoprotein cholesterol; IA-2A; insulinoma-associated protein 2 antibody; IAA, insulin autoantibody; LADA, latent autoimmune diabetes in adults, LDL-C, low-density lipoprotein cholesterol; MG, mean glucose; Scr, serum creatinine; TAR, time above range; TC, total cholesterol; TG, triglyceride; TIR, time in range; TBR, time below range; TPOAb, thyroid peroxidase antibody; UA, uric acid.

When further comparing the clinical characteristics between the LADA and classic T1DM groups (presented in **Table 2**), no significant differences were observed in sex distribution, BMI, TBR, glucose CV, MG, HbA1c, FBG, or daily insulin dose (all *P* > 0.05). However, patients in the LADA group were significantly older (*P* = 0.006) and had a shorter disease duration (*P* < 0.001) compared with those in the classic T1DM group. Regarding glycemic parameters, although no significant differences were found in conventional metrics, CGM-derived metrics including TIR and TAR showed significant differences. Specifically, patients with LADA exhibited higher FCP levels (*P* < 0.001) and increased TIR (*P* = 0.028), accompanied by decreased TAR (*P* = 0.035) compared with the classic T1DM group. Furthermore, autoantibody profiles revealed that IAA (*P* = 0.024) and GADA (*P* < 0.001) levels were significantly higher in the LADA group, and a higher proportion of LADA patients were co-positive for IA-2A and GADA (*P* = 0.038) and IAA and GADA (*P* < 0.001). For biochemical evaluations, patients with LADA demonstrated significantly higher levels of TG (*P* = 0.004), LDL-C (*P* = 0.023), and ALT (*P* = 0.014), alongside lower eGFR levels (*P* = 0.047) than those in the classic T1DM group.

**Table 2:** Clinical characteristics of patients with LADA and classic T1DM.

Variables	Total	LADA	Classic T1DM	*P*
Participants (n)	112	44	68	-
FCP (ng/mL)	0.10 (0.01, 0.37)	0.29 (0.02, 0.52)	0.04 (0.00, 0.24)	< 0.001
Male sex, n (%)	55 (49.11)	26 (59.09)	29 (42.65)	0.089
Age (years)	41.42 ± 18.77	46.98 ± 14.20	37.82 ± 20.51	0.006
BMI (kg/m2)	21.65 ± 3.34	22.12 ± 3.16	21.35 ± 3.43	0.235
Duration of T1DM (years)	6.73 ± 6.77	4.26 ± 4.88	8.33 ± 7.35	< 0.001
TIR (%)	45.67 ± 23.54	51.73 ± 25.64	41.75 ± 21.36	0.028
TAR (%)	53.56 ± 23.86	47.68 ± 25.76	57.37 ± 21.90	0.035
TBR (%)	0.00 (0.00, 1.00)	0.00 (0.00, 1.00)	0.00 (0.00, 1.00)	0.278
Glucose CV (%)	28.46 ± 6.86	27.34 ± 6.44	29.18 ± 7.07	0.167
MG (mmol/L)	10.89 ± 2.21	10.56 ± 2.50	11.11 ± 1.99	0.198
HbA1c (%)	9.43 ± 2.37	9.66 ± 2.57	9.27 ± 2.23	0.410
FBG (mmol/L)	12.71 ± 4.85	13.00 ± 4.98	12.52 ± 4.79	0.610
Daily insulin dose (units/day)	32.22 ± 10.91	30.52 ± 10.28	33.32 ± 11.23	0.186
IA-2A (U/mL)	5.30 (4.84, 37.80)	5.43 (4.77, 42.00)	5.14 (4.88, 11.21)	0.760
IAA (U/mL)	7.63 (6.05, 19.40)	10.02 (6.57, 22.50)	6.95 (5.35, 10.20)	0.024
GADA (U/mL)	74.40 (21.10, 232.00)	115.50(52.12, 280.00)	24.00 (5.51, 127.00)	< 0.001
IA-2A+IAA, n (%)	4 (3.571)	3 (6.818)	1 (1.471)	0.333
IA-2A+GADA, n (%)	18 (16.071)	11 (25.000)	7 (10.294)	0.038
IAA+GADA, n (%)	13 (11.607)	11 (25.000)	2 (2.941)	< 0.001
IA-2A+IAA+GADA, n (%)	4 (3.571)	3 (6.818)	1 (1.471)	0.333
TPOAb (U/mL)	15.35 (11.72, 85.08)	15.00 (10.80, 52.55)	15.70 (12.20, 104.00)	0.368
TG (mmol/L)	1.23 (0.83, 1.60)	1.40 (1.12, 2.05)	1.10 (0.78, 1.48)	0.004
TC (mmol/L)	5.18 ± 1.47	5.50 ± 1.64	4.95 ± 1.30	0.063
LDL-C (mmol/L)	2.94 ± 0.93	3.20 ± 0.96	2.76 ± 0.88	0.023
HDL-C (mmol/L)	1.44 ± 0.43	1.40 ± 0.48	1.46 ± 0.39	0.490
ALT (U/L)	15.00 (11.00, 21.00)	18.00 (13.00, 25.00)	13.50 (10.25, 17.75)	0.014
AST (U/L)	17.00 (14.00, 23.50)	18.00 (15.00, 25.00)	16.00 (14.00, 21.75)	0.218
BUN (mmol/L)	5.95 ± 2.87	6.20 ± 3.44	5.79 ± 2.43	0.477
Scr (umol/L)	62.58 ± 28.47	64.30 ± 16.55	61.45 ± 34.20	0.617
UA (umol/L)	276.00 (222.00, 343.00)	291.00 (237.50, 347.75)	261.50 (204.00, 313.50)	0.063
eGFR (mL/min)	113.00 (99.22, 129.00)	108.90 (99.00, 121.50)	114.90 (102.75, 131.80)	0.047

Data are presented as mean ± SD, median (IQR), or n (%). ALT, alanine aminotransferase; AST, aspartate aminotransferase; BUN, blood urea nitrogen; CV, coefficient of variation; eGFR, estimated glomerular filtration rate; FBG, fasting blood glucose; FCP, fasting C-peptide; GADA, glutamic acid decarboxylase antibody; HbA1c, glycated hemoglobin; HDL-C, high-density lipoprotein cholesterol; IA-2A, insulinoma-associated protein 2 antibody; IAA, insulin autoantibody; LADA, latent autoimmune diabetes in adults; LDL-C, low-density lipoprotein cholesterol; MG, mean glucose; Scr, serum creatinine; TAR, time above range; TC, total cholesterol; TG, triglyceride; TIR, time in range; TBR, time below range; TPOAb, thyroid peroxidase antibody; UA, uric acid.

### 3.2 Univariable associations between residual β-cell function and better glycemic control

**Figure 2:**
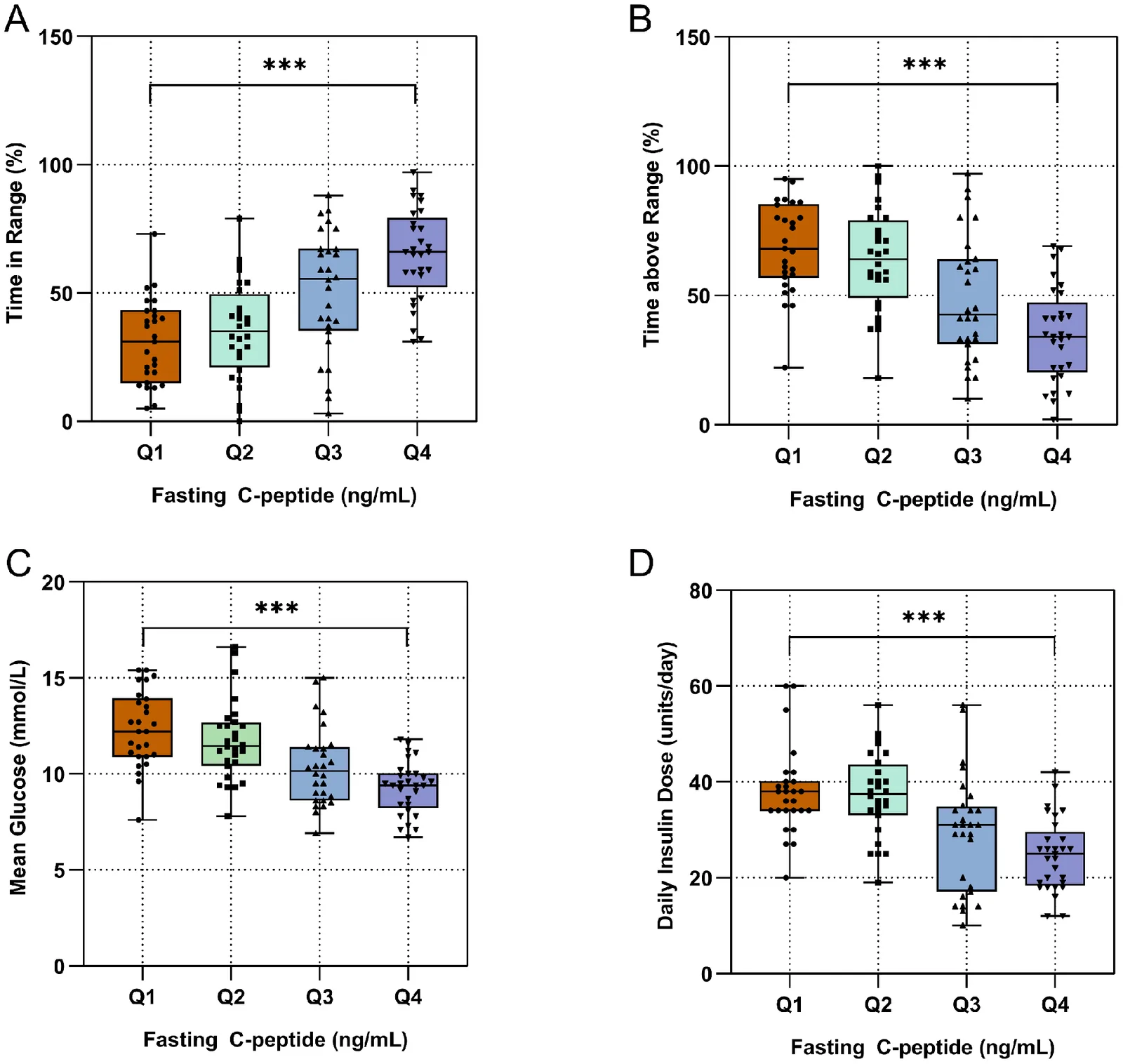
Box plot visualization of glycemic control parameters across quartiles of fasting C-peptide levels. **(A)** Time in range. **(B)** Time above range. **(C)** Mean glucose. **(D)** Daily insulin dose. Intergroup comparisons were performed using one-way ANOVA (overall *P* < 0.001 for all parameters). *Post-hoc* pairwise comparisons were conducted using Tukey’s honestly significant difference test method. *** indicates *P* < 0.001. Fasting C-peptide groups: Q1 (< 0.01 ng/mL), Q2 (0.01–0.09 ng/mL), Q3 (0.09–0.37 ng/mL), and Q4 (> 0.37 ng/mL).

To further visualize the impact of residual islet β-cell function on glycemic control, boxplot analysis was performed (**Figure 2**). Statistically significant overall differences were observed among the four groups in TIR, TAR, MG, and daily insulin dose (one-way ANOVA; F = 17.749, 16.389, 15.150, and 12.936, respectively; all *P* < 0.001). *Post-hoc* Tukey tests demonstrated that the Q4 group exhibited significantly higher TIR (*P* < 0.001) and concurrently lower TAR and MG (both *P* < 0.001) compared with the Q1 group, accompanied by reduced daily insulin requirements (*P* < 0.001).

### 3.3 Correlation between residual β-cell function and clinical parameters

**Figure 3:**
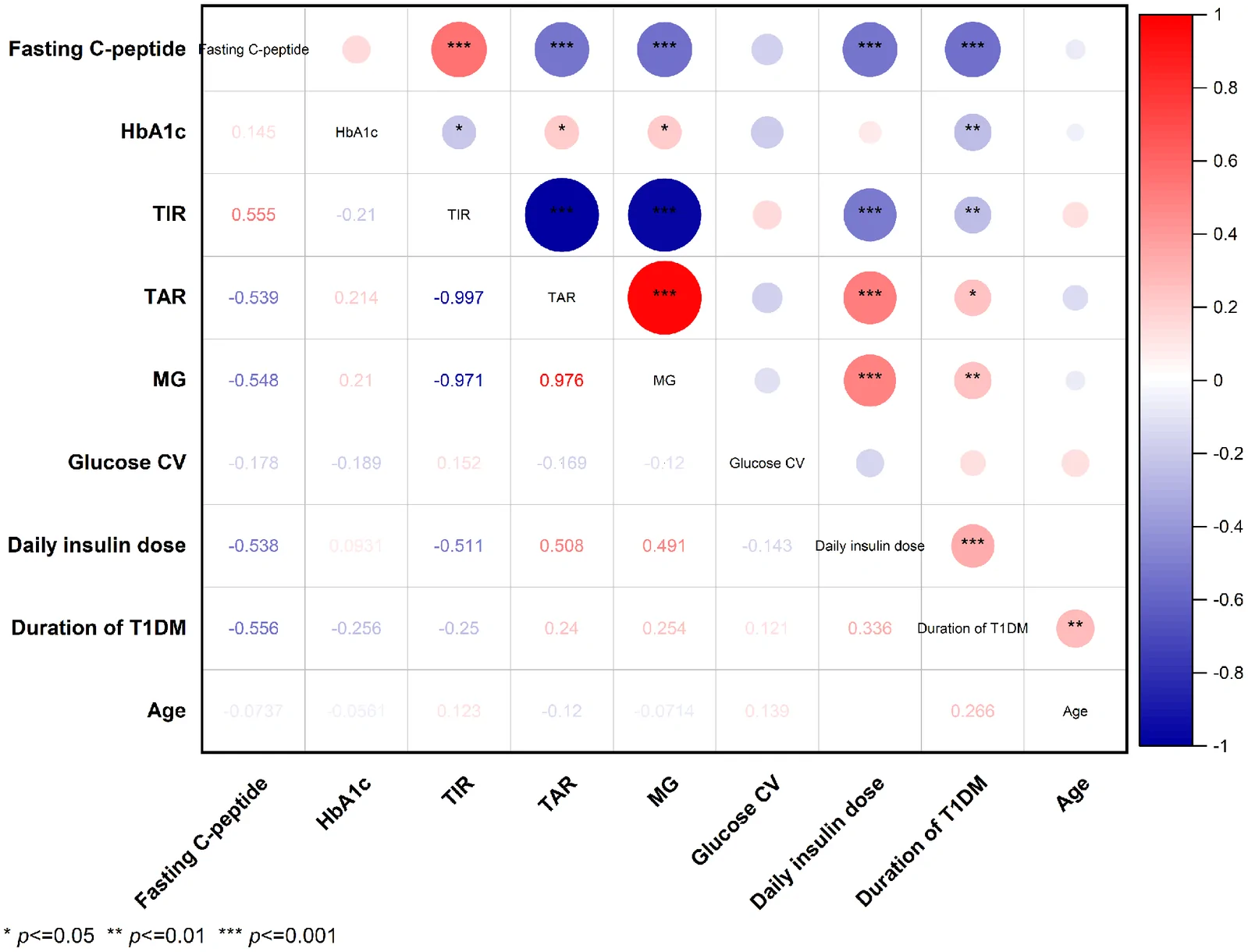
Correlations between fasting C-peptide and clinical parameters using Spearman correlation analysis. CV, coefficient of variation; HbA1c, glycated hemoglobin; MG, mean glucose; TIR, time in range; TAR, time above range.

Given the skewed distribution of FCP (Shapiro-Wilk test, *P* < 0.001), Spearman correlation analysis was employed to evaluate interrelationships among key continuous variables, with results visualized via a correlation matrix heatmap (**Figure 3**). FCP demonstrated a significant positive correlation with TIR (r = 0.555, *P* < 0.001) and significant negative correlations with MG (r = −0.548, *P* < 0.001) and TAR (r = −0.539, *P* < 0.001), suggesting that preserved residual β-cell function contributes to improved glycemic stability. Additionally, FCP showed moderate negative correlations with disease duration (r = −0.556, *P* < 0.001) and daily insulin dose (r = −0.538, *P* < 0.001). Glycemic control indicators (TIR, MG, HbA1c, TAR) also exhibited highly consistent intrinsic intercorrelations.

### 3.4 Restricted cubic spline analysis of the dose–response relationships between fasting C-peptide and CGM-derived metrics

**Figure 4:**
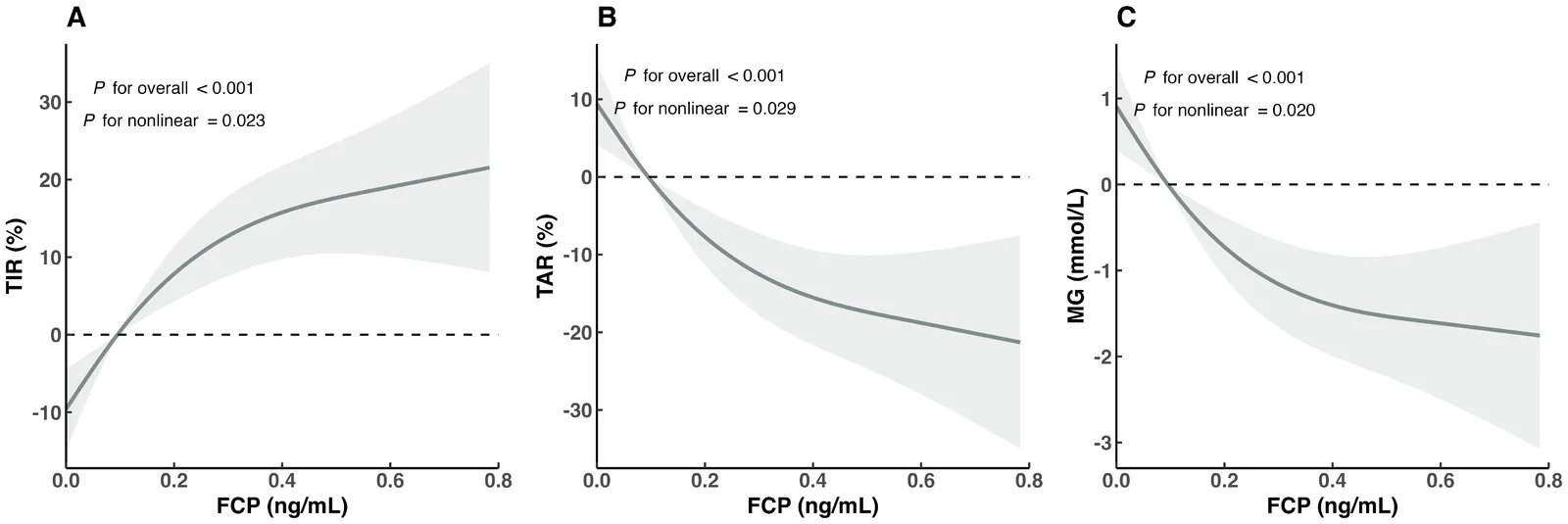
Restricted cubic spline (RCS) curves illustrating the dose-response relationships between fasting C-peptide (FCP) and CGM-derived metrics in the overall population. **(A)** Non-linear relationship between FCP and TIR (*P* for overall < 0.001, *P* for nonlinearity = 0.023). **(B)** Non-Linear relationship between FCP and TAR (*P* for overall < 0.001, *P* for nonlinearity = 0.029). **(C)** Non-linear relationship between FCP and MG (*P* for overall < 0.001, *P* for nonlinearity = 0.020). Solid gray lines indicate multivariable-adjusted regression estimates, and shaded areas represent the 95% confidence intervals based on multivariable linear regression Model 4 (adjusted for sex, age, disease duration, BMI, daily insulin dosage, and glucose coefficient of variation). Dashed lines represent the reference line. MG, mean glucose; TAR, time above range; TIR, time in range.

To explore the potential non-linear dose-response relationships between FCP levels and CGM-derived metrics (TIR, TAR, and MG) in the entire study population, RCS regression models adjusted for Model 4 covariates were constructed (**Figure 4**). The analysis revealed a significant non-linear relationship between FCP and TIR (*P* for overall < 0.001, *P* for nonlinearity = 0.023; **Figure 4A**). The curve demonstrated that TIR increased sharply with the elevation of FCP at lower ranges and began to plateau as FCP reached higher levels, suggesting a diminishing marginal benefit of residual β-cell function beyond a certain threshold. Conversely, a significant non-linear inverse association was observed between FCP and TAR (*P* for overall < 0.001, *P* for nonlinearity = 0.029; **Figure 4B**), displaying a steep reduction in TAR at lower FCP levels that flattened out as FCP levels increased. FCP exhibited a significant non-linear association with MG (*P* for overall < 0.001, *P* for nonlinearity = 0.020; **Figure 4C**), characterized by a steep decline in MG at lower FCP levels, which gradually decelerated and flattened out at higher FCP levels. Although a statistically significant nonlinear component was detected for TIR, TAR, and MG, the dose-response curves exhibited overall monotonic trends. Therefore, multivariable linear regression analyses were performed to quantify the average independent associations between FCP and CGM-derived metrics across the observed range after comprehensive adjustment for potential confounders.

### 3.5 Multivariable linear regression analyses of the associations between fasting C-peptide and CGM-derived metrics

To further control for confounding factors and confirm the independent predictive value of FCP across different CGM parameters, multivariable linear regression models were constructed (**Table 3**). For TIR, the analysis demonstrated that FCP remained significantly and positively associated with TIR. In Model 1 (crude analysis), each 1 ng/mL increase in FCP was associated with a 19.83 percentage point increase in TIR (95% *CI*: 10.66–29.01, *P* < 0.001). This association retained statistical significance after adjustment for sex, age, disease duration, and BMI (Model 2: *β* = 20.25, 95% *CI*: 10.41–30.09, *P* < 0.001). Upon further adjustment for daily insulin dosage, the effect estimate attenuated substantially (Model 3: *β* = 12.86, 95%*CI*: 3.31–22.42, *P* = 0.009), suggesting that insulin treatment intensity represents an important confounding factor. In the final model (Model 4), which additionally adjusted for glucose CV, the effect size of FCP increased modestly (*β* = 15.75, 95% *CI*: 5.96–25.53, *P* = 0.002). For TAR, FCP consistently demonstrated a robust and independent negative association across all multivariable models. In Model 1, each 1 ng/mL increase in FCP was associated with a 46.56 percentage point reduction in TAR (*β* = -46.56, 95% CI: -61.55 to -31.58, *P* < 0.001). This inverse association remained highly significant in the fully adjusted model (Model 4: β = -40.74, 95% CI: -58.73 to -22.74, *P* < 0.001), highlighting that preserved β-cell function substantially alleviates exposure to hyperglycemia. For MG, FCP consistently exhibited a robust and independent negative association across all four models. In the fully adjusted model (Model 4), per 1 ng/mL increase in FCP was independently associated with a 3.56 mmol/L decrease in MG (*β* = -3.56, 95% *CI*: -5.30 to -1.82, *P* < 0.001), underscoring the critical role of residual islet function in lowering overall glucose levels.

**Table 3:** Multivariable linear regression analysis of the association between fasting C-peptide and CGM-derived metrics.

Variables	Model1		Model2		Model3		Model4	
	β (95%CI)	*P*	β (95%CI)	*P*	β (95%CI)	*P*	β (95%CI)	*P*
Model-TIR	19.83 (10.66 ~ 29.01)	< 0.001	20.25 (10.41 ~ 30.09)	< 0.001	12.86 (3.31 ~ 22.42)	0.009	15.75 (5.96 ~ 25.53)	0.002
Model-TAR	-46.56 (-61.55 ~ -31.58)	< 0.001	-49.46 (-66.06 ~ -32.86)	< 0.001	-34.16 (-52.04 ~ -16.27)	< 0.001	-40.74 (-58.73 ~ -22.74)	< 0.001
Model-MG	-4.04 (-5.46 ~ -2.63)	< 0.001	-4.32 (-5.90 ~ -2.75)	< 0.001	-2.99 (-4.70 ~ -1.27)	< 0.001	-3.56 (-5.30 ~ -1.82)	< 0.001

β represents the estimated change in the outcome variable (TIR, TAR, or MG) per 1 ng/mL increase in fasting C-peptide. CI: confidence interval. Model 1, unadjusted analyses; Model 2, adjusted for sex, age, disease duration, and BMI; Model 3, additionally adjusted for daily insulin dosage; Model 4, further adjusted for glucose coefficient of variation. MG, mean glucose; TAR, time above range; TIR, time in range.

### 3.6 Subgroup analyses according to diabetes subtype

**Figure 5:**
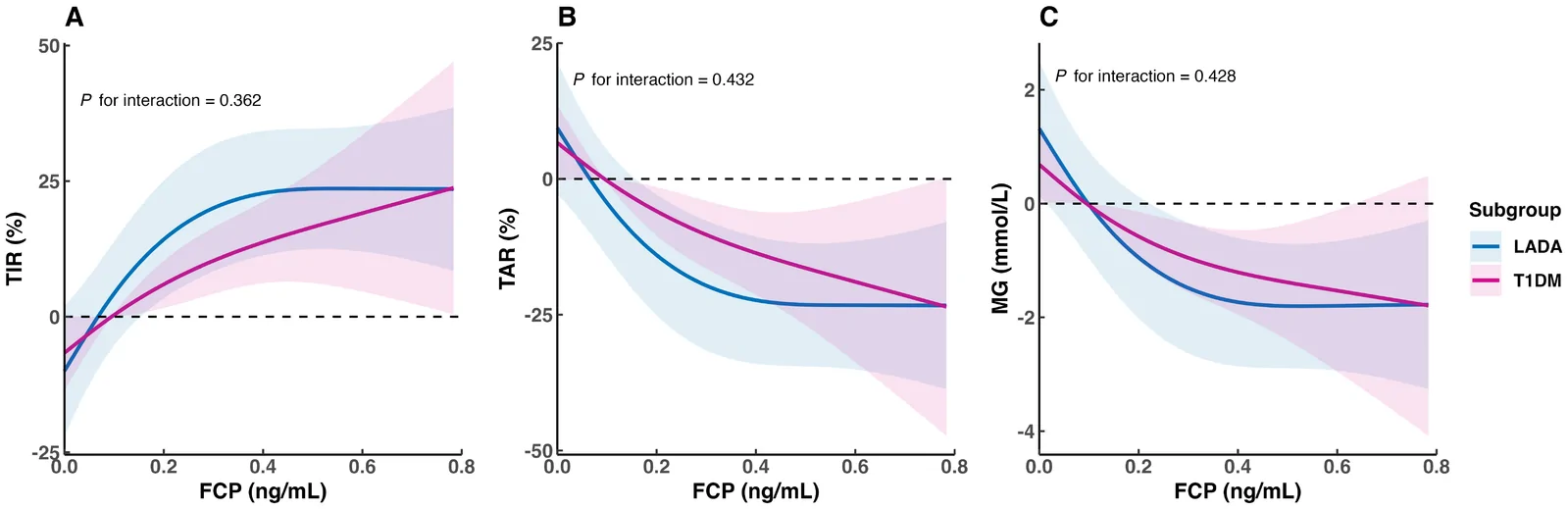
Subgroup restricted cubic spline (RCS) analysis comparing the dose-response relationships between fasting C-peptide (FCP) and CGM-derived metrics between LADA and classic T1DM groups. **(A)** Subgroup curves for TIR (*P* for interaction = 0.362). **(B)** Subgroup curves for TAR (*P* for interaction = 0.432). **(C)** Subgroup curves for MG (*P* for interaction = 0.428). Blue curves/shaded areas represent the LADA group, and pink curves/shaded areas represent the classic T1DM group. Shaded areas represent 95% confidence intervals adjusted for covariates in Model 4. Dashed lines represent the reference line. LADA, latent autoimmune diabetes in adults; MG, mean glucose; T1DM, type 1 diabetes mellitus; TIR, time in range; TAR, time above range.

In response to the clinical heterogeneity between LADA and classic T1DM, we performed subgroup RCS analyses to test whether the dose-response relationships between FCP and CGM metrics differed between these two sub-phenotypes (**Figure 5**). The multi-panel spline plots demonstrated that the patterns of FCP’s impact on TIR, TAR, and MG were comparable between the LADA and classic T1DM groups. Specifically, no significant interactions were detected between FCP and diabetes subtype for TIR (*P* for interaction = 0.362; **Figure 5A**), TAR (*P* for interaction = 0.432; **Figure 5B**), or MG (*P* for interaction = 0.428; **Figure 5C**). These findings suggest that although LADA and classic T1DM have distinct clinical and onset characteristics, the independent protective effect of residual β-cell function on stabilizing glucose levels and improving CGM-derived metrics remains universal across both subtypes.

### 3.7 Sensitivity analysis

A series of sensitivity analyses were performed using the fully adjusted model to evaluate the robustness of our primary findings (**Table 4**). First, when FCP was treated as a quartile-based categorical variable, significant linear trends across increasing FCP quartiles were confirmed for TIR, TAR, and MG (*P*_for trend_ < 0.001 for all). Second, after entirely excluding LADA patients, higher FCP levels remained strongly and independently associated with increased TIR (*β* = 45.86, *P* < 0.001), decreased TAR (β = -55.36, *P* < 0.001), and decreased MG (*β* = -3.86, *P* = 0.001). Third, excluding patients with a diabetes duration ≧10 years yielded highly consistent and robust associations for both TIR (*β* = 39.20, *P* < 0.001), TAR (β = -38.60, *P* < 0.001), and MG (*β* = -3.26, *P* = 0.003). Taken together, these findings firmly established the stability of our primary conclusions.

**Table 4:** Sensitivity analyses evaluating the independent associations of fasting C-peptide with CGM metrics.

Analysis approach	Model-TIR		Model-TAR		Model-MG	
	β (95%CI)	*P*	β (95%CI)	*P*	β (95%CI)	*P*
FCP as a categorical variable	–	**-**	–	–	–	–
Q1(Reference)	–	**-**	–	–	–	–
Q2	5.90 (-3.79 ~ 15.60)	0.236	-6.42 (-16.29 ~ 3.45)	0.205	-0.80 (-1.74 ~ 0.15)	0.100
Q3	19.66 (8.86 ~ 30.46)	<0.001	-19.68 (-30.67 ~ -8.70)	<0.001	-2.03 (-3.08 ~ -0.99)	<0.001
Q4	31.39 (19.44 ~ 43.34)	<0.001	-31.36 (-43.52 ~ -19.19)	<0.001	-2.99 (-4.15 ~ -1.83)	<0.001
*P*_for trend_	–	<0.001	–	<0.001	–	<0.001
Excluding LADA	45.86 (22.89 ~ 68.83)	<0.001	-55.36 (-77.96 ~ -32.77)	<0.001	-3.86 (-6.10 ~ -1.63)	0.001
Excluding patients with a diabetes duration ≥ 10 years	39.20 (17.71 ~ 60.70)	<0.001	-38.60 (-60.36 ~ -16.83)	<0.001	-3.26 (-5.32 ~ -1.19)	0.003

Data are presented as regression coefficients (β) with 95% confidence intervals (CIs) and *P*-values derived from multivariable linear regression. All sensitivity models were constructed based on the fully adjusted model (equivalent to Model 4 in **Table 3**). Sex, age, BMI, daily insulin dose, and glucose CV were mutually adjusted across all models. Additionally, diabetes duration was adjusted for in the categorical fasting C-peptide model and the model excluding LADA patients, but was omitted from the model restricted to patients with a duration <10 years.

## 4 Discussion

This study evaluated the relationship between residual islet β-cell function, assessed by FCP, and CGM-derived metrics in patients with T1DM. The results demonstrated that higher FCP levels were associated with increased TIR, decreased TAR, and lower MG. Due to the heterogeneity of LADA and classic T1DM in genetic background, autoimmune process, and environmental factors, a spectrum of clinical phenotypes with variable degrees of insulin deficiency and insulin resistance is observed (15). This study further compared the clinical differences between LADA and classic T1DM. In our study population, the LADA group was significantly older and had shorter disease duration. Although traditional glycemic metrics showed no significant differences, CGM-derived metrics TIR and TAR were significantly different. Compared with the classic T1DM group, the LADA group had higher FCP levels, increased TIR, and decreased TAR. Subgroup analysis revealed consistent associations between FCP and CGM-derived metrics in both classic T1DM and LADA, indicating that the protective effect of residual β-cell function on improving CGM-derived metrics is universally present in both subtypes. Additionally, shorter disease duration correlated with higher FCP levels and reduced daily insulin requirements.

T1DM is an autoimmune disorder characterized by progressive destruction of pancreatic β-cells and consequent insulin deficiency (16). However, the use of insulin as a biomarker to assess residual β-cell function is inherently problematic due to its short half-life and variable first-pass hepatic extraction (17). Compared with insulin, C-peptide exhibits superior stability and a substantially longer half-life, establishing it as the preferred biomarker for evaluating endogenous insulin secretory capacity. C-peptide serves as an ideal indicator for assessing residual β-cell activity, with longitudinal studies having confirmed its associations with HbA1c, risk of microvascular complications, and frequency of hyperglycemic episodes (18). Consequently, C-peptide is frequently employed as the primary outcome measure in interventional trials for T1DM (19). While previous studies have suggested rapid decline in insulin secretion following T1DM diagnosis (20), other investigations have demonstrated a more gradual decrease in serum C-peptide levels, with preserved insulin secretory function observed in a subset of patients (21). The DCCT demonstrated that a considerable proportion of adults maintain β-cell function several years after T1DM onset, with detectable C-peptide observable in some individuals even after 50 years of disease duration (22). Residual β-cell function may confer multiple clinical benefits, including reduced hypoglycemia risk (4), improved metabolic control as reflected by HbA1c, decreased exogenous insulin requirements, and attenuated risk of long-term complications (23). Consistent with these findings, our study identified detectable C-peptide levels in a subset of patients with T1DM, with levels inversely correlated with disease duration; patients with favorable C-peptide profiles typically had disease duration of less than 3 years. Additionally, C-peptide levels were inversely associated with daily insulin dosage.

Previous studies employing mixed-meal tolerance tests have established the importance of residual β-cell function in maintaining glycemic control in T1DM. Endogenously secreted insulin enters the portal circulation directly, enabling the liver to store postprandial glucose as glycogen—a physiological advantage that subcutaneous insulin administration cannot fully replicate (24). Preserved β-cell function also dampens inappropriate glucagon release during hyperglycemia, thereby attenuating hepatic glucose output and preventing a self-perpetuating cycle of metabolic deterioration (25). Additionally, even modest residual C-peptide levels are associated with reduced glycemic variability, fewer hypoglycemic events, and lower HbA1c (26). However, owing to substantial glycemic variability in T1DM, HbA1c fails to fully capture glucose fluctuations. The widespread adoption of CGM technology has addressed these limitations of conventional glucose monitoring by enabling comprehensive recording of daily glucose profiles and providing a more complete and accurate representation of overall glycemic variability. Consequently, CGM-derived metrics—including TIR, TAR, TBR, and glucose CV—have emerged as novel critical metrics for clinical glycemic assessment. TIR, in particular, serves as an important indicator for evaluating glycemic variation and has been associated with both microvascular and macrovascular complications of diabetes (27). Prior research has demonstrated correlations between residual β-cell function and CGM-derived metrics in patients with T1DM. Recent clinical evidence has also demonstrated that intermittently scanned CGM systems can reliably capture key glucose metrics in T1DM (28). A US-based study conducted in highly selected participants revealed associations between residual β-cell function—assessed via mixed-meal tolerance tests—and CGM-derived metrics (24). This study extends these findings by confirming that FCP is associated with prolonged TIR, shortened TAR, and reduced MG. Moreover, in multivariable linear regression analyses adjusting for sex, age, BMI, insulin dosage, and glycemic CV, FCP remained robustly and independently positively associated with TIR, and negatively associated with TAR and MG. Despite the distinct clinical and pathogenetic characteristics of LADA and classic T1DM, the protective effect of residual β-cell function on glycemic stability and improved CGM-derived metrics is universally observed in both subtypes.

The present study demonstrated that FCP was associated with longer TIR, shorter TAR, and lower MG, indicating that residual pancreatic β-cell function plays a critical role in reducing overall glycemic exposure and maintaining favorable glucose homeostasis in T1DM. These findings underscore the impact of preserved C-peptide secretion on glycemic control, manifesting as attenuated glycemic variability and reduced hyperglycemic burden. Despite universal insulin pump therapy among all participants, our results suggest that intensified efforts to identify novel strategies for protecting β-cell function are warranted to achieve superior glycemic homeostasis (29). This contention is supported by a previous cohort study demonstrating that even minimal residual C-peptide confers clinical benefit in T1DM, highlighting that pancreatic islet preservation or restoration represents a crucial therapeutic target (30). In this study, RCS analysis similarly revealed that glycemic control metrics including TIR, TAR, and MG improved dramatically at lower FCP concentrations. Furthermore, sensitivity analyses demonstrated that after excluding patients with LADA, the association between FCP and CGM-derived variables remained even stronger in classic T1DM, which exhibited lower mean FCP levels. Therefore, maintaining endogenous β-cell function, such as promoting β-cell regeneration (31), is an important goal in the management of T1DM. The DCCT demonstrated that intensive treatment in T1DM helps sustain endogenous insulin secretion, and that intensive glycemic control significantly delays C-peptide decline and protects residual β-cell function in newly diagnosed patients (32). CGM provides an essential monitoring tool for intensive management of T1DM, particularly when combined with insulin pump therapy, enabling fine-tuning of insulin dosing across time intervals based on ambulatory glucose profiles to maintain glucose homeostasis, reduce glycemic variability, and minimize both hyperglycemic and hypoglycemic events. The absence of significant differences in TBR across C-peptide groups in our study likely reflects the relatively short CGM wear duration and the low overall frequency of hypoglycemic episodes in this cohort.

While prior studies have consistently linked residual β-cell function to glycemic control in T1DM, this study further delineates the dose-dependent relationship between FCP and CGM-derived metrics in both classic T1DM and LADA. However, this study has several limitations. First, the sample size was relatively modest. Second, as not all participants underwent comprehensive screening for diabetic microvascular and macrovascular complications, we were unable to examine the relationship between C-peptide and these complications. Third, the 7-day CGM wear time was relatively short. Future research should expand separate cohorts, extend CGM duration, and integrate CGM metrics with C-peptide levels to guide precision therapy—including individualized lifestyle prescriptions—to achieve sustained euglycemia and preserve β-cell function.

## 5 Conclusions

Residual pancreatic β-cell function in T1DM, encompassing both classic T1DM and LADA, is associated with favorable CGM-derived metrics, including increased TIR, decreased TAR, and lower MG. Disease duration exhibits an inverse relationship with FCP levels, while preserved C-peptide secretion is associated with lower daily insulin requirements. These findings underscore that protecting residual β-cell function in T1DM contributes to glycemic stability and reduced dependence on exogenous insulin.

## Data Availability

The raw data supporting the conclusions of this article will be made available by the authors, without undue reservation.
